# Chemoprotective Effect of Taurine on Potassium Bromate-Induced DNA Damage, DNA-Protein Cross-Linking and Oxidative Stress in Rat Intestine

**DOI:** 10.1371/journal.pone.0119137

**Published:** 2015-03-06

**Authors:** Mir Kaisar Ahmad, Aijaz Ahmed Khan, Shaikh Nisar Ali, Riaz Mahmood

**Affiliations:** 1 Department of Biochemistry, Faculty of Life Sciences, Aligarh Muslim University, Aligarh, Uttar Pradesh, India; 2 Department of Anatomy, Faculty of Medicine, J. N. Medical College, Aligarh Muslim University, Aligarh, Uttar Pradesh, India; Indian Institute of Integrative Medicine, INDIA

## Abstract

Potassium bromate (KBrO_3_) is widely used as a food additive and is a major water disinfection by-product. It induces multiple organ toxicity in humans and experimental animals and is a probable human carcinogen. The present study reports the protective effect of dietary antioxidant taurine on KBrO_3_-induced damage to the rat intestine. Animals were randomly divided into four groups: control, KBrO_3_ alone, taurine alone and taurine+ KBrO_3_. Administration of KBrO_3_ alone led to decrease in the activities of intestinal brush border membrane enzymes while those of antioxidant defence and carbohydrate metabolism were also severely altered. There was increase in DNA damage and DNA-protein cross-linking. Treatment with taurine, prior to administration of KBrO_3_, resulted in significant attenuation in all these parameters but the administration of taurine alone had no effect. Histological studies supported these biochemical results showing extensive intestinal damage in KBrO_3_-treated animals and greatly reduced tissue injury in the taurine+ KBrO_3_ group. These results show that taurine ameliorates bromate induced tissue toxicity and oxidative damage by improving the antioxidant defence, tissue integrity and energy metabolism. Taurine can, therefore, be potentially used as a therapeutic/protective agent against toxicity of KBrO_3_ and related compounds.

## Introduction

Potassium bromate (KBrO_3_) is a food additive that is extensively used as a maturing agent for flour and as a dough conditioner. It is also used in cosmetics and is a component of permanent hair weaving solutions. Disinfection of drinking water by ozonation, which has emerged as a promising alternative to chlorination since it does not result in the production of hazardous agents like trihalomethanes, also generates bromate as a by-product [1]. During ozonation, the bromide contained in water naturally is oxidized to bromate which is thus frequently detected in tap and even bottled water. Exposure to KBrO_3_ results in multiple organ toxicity with kidney being the primary target organ of this compound. KBrO_3_ has been shown to alter gene expression in renal tissues and chronic administration of KBrO_3_ induces carcinomas in rats, hamsters and mice [2–4]. Bromate is now considered as a probable human carcinogen and a complete carcinogen in animals. Increased production of reactive oxygen species (ROS) and free radicals has been implicated in mediating KBrO_3_-induced toxicity. These radicals can cause extensive tissue damage by reacting with macromolecules like proteins, nucleic acids and membrane lipids which causes an imbalance in homeostasis and leads to tissue injury [2,5,6]. Supporting the involvement of ROS in its action, several antioxidants (AO) have been shown to ameliorate the bromate-induced multiple organ toxicity [7–11].

Taurine (2-aminoethanesulfonic acid) is a conditionally essential amino acid found in large concentrations in all mammalian tissues and accounts for approximately 0.1% of total human body weight. It is present in various foods like eggs, milk and is especially abundant in seafood and meat. Taurine is involved in a number of crucial physiological processes including modulation of calcium flux and neuronal excitability, osmoregulation, detoxification, membrane stabilization, reproduction and immunity [12]. It is essential for the development and survival of mammalian cells, particularly those of the cerebellum, retina and kidney [12,13]. Taurine is also an AO and a potent scavenger of the hydroxyl radical suggesting that it may be useful in treating oxygen radical mediated toxicity [14]. Taurine protects tissues from various pathological conditions resulting from free radicals generated upon exposure to various xenobiotics [15–21].

We have recently shown that administration of KBrO_3_ to rats induces oxidative stress (OS) and lowers the activities of several enzymes in the intestinal brush border membrane (BBM). It causes alterations in the activities of various antioxidant and metabolic enzymes and damages the intestinal DNA [5,6]. In the present work, we have used taurine to attenuate the KBrO_3_-induced intestinal damage using rats as the animal model. This was done in view of the effectiveness of taurine in mitigating toxicities involving ROS and OS. Our results show that taurine is an effective chemoprotective agent in attenuating bromate-induced gastrointestinal damage.

## Materials and Methods

Adult male rats of Wistar strain weighing 150 to 200 g were used in all the experiments. The study was approved by an Institutional Animals Ethical Committee (IAEC) of Aligarh Muslim University that monitors research involving animals. Animals were stabilized for 1 week prior to the experiment on standard pellet rat diet with free access to water. Solutions of taurine and KBrO_3_ were prepared in drinking water and given orally (by gavage) to animals. The animals were randomly divided into four groups with six rats in each group. Group I (control) was given suitable volume of drinking water by gavage, group II (KBrO_3_ alone) was given a single dose of KBrO_3_ at 100 mg/kg body weight while group III (taurine alone) was given taurine at 100 mg/kg body weight/day for five days. Animals in group IV (taurine+KBrO_3_) were first given taurine for five days at 100 mg/kg body weight/day. Then 6 h after the last dose of taurine they were administered single dose of KBrO_3_ at 100 mg/kg body weight.

The animals were sacrificed 48 h after the last treatment under light ether anaesthesia. The entire small intestine was removed, flushed with ice-cold saline and slit open. The mucosa was gently scraped with a glass slide and used for the preparation of brush border membrane vesicles (BBMV) and homogenates. The experiment was planned such that all animals were sacrificed on the same day. Animals had free access to water and food throughout the duration of the experiment.

### Preparation of BBMV and mucosal homogenates

BBMV and mucosal homogenates were prepared exactly as described by Farooq et al. [22] and stored at −20°C until further analysis. Protein concentration in homogenates and BBMV was determined by the Folin phenol reagent using bovine serum albumin as standard [23].

### BBM enzymes

The BBM enzymes were assayed in intestinal homogenates and BBMV as described previously [5]. Briefly, the activity of alkaline phosphatase (AP) was determined at pH 10.5 using p-nitrophenyl phosphate as substrate while γ-glutamyl transferase (GGT) and leucine aminopeptidase (LAP) were assayed using γ-glutamyl p-nitroanilide and L-leucine p-nitroanilide as substrates, respectively. Sucrase was assayed by determining the reducing sugars formed upon hydrolysis of sucrose after reaction with 3,5-dinitrosalicylic acid. The kinetic parameters K_M_ and V_max_ were determined by assaying the enzymes at different substrate concentrations in isolated BBMV and analyzing the data by double reciprocal Lineweaver-Burk plots.

### Acid phosphatase (ACP) and total adenosine 5’-triphosphatase (ATPase)

The activity of lysosomal marker enzyme ACP was determined in intestinal homogenates at pH 4.5 using p-nitrophenyl phosphate as substrate [24]. Total ATPase was assayed by the method of Konkel et al. [25]. Briefly, the intestinal homogenates were incubated with 2 mM adenosine 5’-triphosphate (ATP) in 1.5 ml reaction volume at 37°C for 15 min and the reaction was terminated by addition of 0.2 ml of 30% tricarboxylic acid. The samples were centrifuged at 4,000 rpm and inorganic phosphate released was measured in protein free supernatant using ferrous ammonium molybdate as coloring reagent.

### Enzymes involved in free radical scavenging

Cu-Zn superoxide dismutase (SOD) was assayed by following the inhibition of auto-oxidation of pyrogallol [26] and catalase (CAT) from the conversion of hydrogen peroxide (H_2_O_2_) to water [27]. Glutathione reductase (GR) was assayed from the conversion of nicotinamide adenine dinucleotide phosphate reduced (NADPH) to nicotinamide adenine dinucleotide phosphate (NADP^+^) during the reduction of oxidised glutathione to reduced glutathione (GSH) [28]. Glutathione-S-transferase (GST) activity was determined by using 1-chloro-2, 4-dinitrobenzene as substrate [29] and thioredoxin reductase (TR) by following the yellow color formed at 410 nm upon reduction of 5,5’-dithiobis-2-nitrobenzoic acid by NADPH [30]. Glutathione peroxidase (GPx) was assayed from the conversion of NADPH to NADP^+^ at 340 nm in the presence of oxidised glutathione [31].

### Enzymes of carbohydrate metabolism

Enzyme activities involving oxidation of NADH or reduction of NADP^+^ were determined in mucosal homogenates from absorbance changes at 340 nm. A molar extinction coefficient of 6.22×10^3^M^−1^ cm^−1^ was used for calculating the concentrations of NADH/NADPH. The activities of lactate dehydrogenase (LDH), malate dehydrogenase (MDH), malic enzyme (ME), glucose 6-phosphatase (G6P) and fructose 1,6-bisphosphatase (FBP) were assayed as described by Khundmiri et al. [32]. Glucose 6-phosphate dehydrogenase (G6PD) activity was measured by glucose 6-phosphate dependent reduction of NADP^+^ to NADPH [33].

### Thiobarbituric acid reactive substances (TBARS), carbonyl content, GSH, total sulfhydryl (SH) and H_2_O_2_ levels

Lipid peroxidation (LPO) was determined from the level of TBARS [34]. Protein carbonyl content was determined after reaction with 2,4-dinitrophenyl hydrazine [35]. SH groups and GSH were measured from the yellow color produced after their reaction with 5,5’-dithiobis-2-nitrobenzoic acid [36]. H_2_O_2_ levels were determined using xylenol orange as color reagent in the presence of 100 mM sorbitol [37].

### Histology

Sections of the duodenum were fixed in 10% formalin, cut into 10 μm sections and stained with hematoxylin and eosin by routine procedure [38]. The slides were examined under a microscope (Olympus BX40, Japan) at 400X magnification.

### Comet Assay

The intestinal mucosa was removed immediately after sacrifice of animals and transferred into the Roswell Park Memorial Institute (RPMI) medium containing 1 mM EDTA. The solutions were sieved by muslin cloth into Petri dishes to collect the cell suspension. Single cell gel electrophoresis (Comet assay) was performed in alkaline conditions as described by Singh et al. [39]. The DNA was stained by ethidium bromide and visualized under a CX41 fluorescence microscope (Olympus, Japan). The comets were scored at a magnification of 100X and images of 50 cells (25 from each replicate slide) for each sample were scored. Comet tail length (migration of DNA from the nucleus in μm) was chosen as the parameter to assess nuclear DNA damage and was automatically generated by the Komet 5.5 (USA) image analysis system.

### DNA fragmentation and DNA-protein cross-linking (DPC)

Quantitation of DNA fragmentation was done by the colorimetric diphenylamine assay [40]. The intestinal homogenates were mixed with equal volume of buffer containing 20 mM Tris-HCl, 20 mM ethylenediaminetetracetate (EDTA), 0.5% Triton X-100, pH 7.5, and centrifuged at 15,000 rpm for 15 min at 4°C to separate intact DNA in the pellet from fragmented/damaged DNA in the supernatant fraction. Perchloric acid (final concentration 0.5 M) was added to the pellet and supernatant samples which were heated at 90°C for 15 min and then centrifuged to remove precipitated proteins. The resulting supernatants, whether containing whole or fragmented DNA, were treated with 58.7 mM diphenylamine for 16–20 h at room temperature in dark and the absorbance was recorded at 600 nm. DNA fragmentation was expressed as the percentage of fragmented DNA to total DNA.

DPC was determined by preparing10% mucosal homogenate in buffer containing 2% SDS, 20 mM Tris-HCl, 20 mM EDTA, pH 7.5 and heating at 65°C for 10 min. Then 1.5 ml of this homogenate was mixed with 0.5 ml of 0.2 M KCl, 20 mM Tris-HCl, pH 7.5, passed five times through a 21-gauge needle and centrifuged. The DNA-protein cross-links were then determined in the supernatants exactly as described by Zhitkovich and Costa [41].

### Statistical analysis

All data are expressed as mean ± standard error of mean (SEM). Analysis of variance was used in combination with Post-Hoc test (Bonferroni comparison test) using GraphPad InStat 3.0. (USA) to evaluate the data by comparing the results of treatment groups to control group. All differences of p < 0.05 were considered significant. All experiments were done at least three times to document reproducibility.

## Results

The effect of oral treatment with taurine on KBrO_3_-induced DNA damage, alterations in BBM enzymes, carbohydrate metabolism and the AO status of rat intestine were studied. The doses of taurine (100 mg/kg/day for 5 days) and KBrO_3_ (single dose of 100 mg/kg body weight) administered to animals were those which have been previously used by us and other workers [5,6,17,18]. Both taurine and KBrO_3_ were given orally to the animals, rather than intraperitoneally or subcutaneously, to simulate real exposure and intake of these agents. Animals in all four groups were sacrificed 48 h after the treatments, and their small intestines were removed. This time interval was selected since our previous work has shown that KBrO_3_-induced intestinal changes were maximum 48 h after its administration (Ahmad et al., 2012 and 2013). Intestinal mucosal homogenates and BBMV were prepared and used in the determination of several biochemical parameters.

### BBM enzymes

Four BBM enzymes (AP, LAP, GGT, and sucrase) were assayed in mucosal homogenates and BBMV prepared from animals in the four groups. Treatment of rats with KBrO_3_ alone resulted in significant decline in the activities of all these enzymes, both in homogenates and BBMV, compared to the control group (Tables 1 and 2). AP and GGT were the enzymes most affected by treatment with KBrO_3_, both in the homogenates and BBMV. Pre-treatment with taurine significantly ameliorated the KBrO_3_-induced changes by bringing the enzyme activities near control values. Administration of taurine alone did not have a significant effect on enzyme activities which were similar to the controls.

**Table 1:** Effect of taurine pre-treatment on KBrO_3_-induced changes in the activities of BBM enzyme in the intestinal homogenates.

	Control	KBrO_3_ alone	Taurine alone	Taurine+KBrO_3_
LAP	3.76±0.29	1.89±0.08*	3.81±0.22^ǂ^	3.08±0.18 * †
AP	2.37±0.19	1.01±0.09*	2.40±0.18^ǂ^	2.05±0.11 * †
GGT	2.26±0.15	0.98±0.07*	2.34±0.12^ǂ^	1.92±0.09 * †
Sucrase	27.12±2.77	13.11±1.12*	26.65±2.89^ǂ^	23.14±2.06 * †

Results are mean ± SEM of six different preparations. Specific activities of enzymes are in μmoles/mg protein/hour.

* Significantly different from control.

† Significantly different at p < 0.05 from KBrO_3_-treated group.

ǂ Significantly different at p < 0.05 from KBrO_3_ and taurine+KBrO_3_ treated group.

LAP: leucineaminopeptidase; AP: alkaline phosphatase; GGT: γ-glutamyltransferase.

**Table 2:** Effect of taurine pre-treatment on KBrO_3_-induced changes in the activities of BBM enzymes in isolated BBMV.

	Control	KBrO_3_ alone	Taurine alone	Taurine+KBrO_3_
LAP	30.96±3.29	15.86±2.08*	30.48±3.17^ǂ^	24.84±2.74* ^†^
AP	20.14±2.37	9.16±1.01*	20.01±2.28^ǂ^	17.42±2.01* ^†^
GGT	18.48±1.89	8.15±0.97*	18.18±1.97^ǂ^	15.55±1.28* ^†^
Sucrase	230.87±10.97	109.93±7.54*	232.11±10.21^ǂ^	196.79±7.64* ^†^

Results are mean ± SEM of four different preparations. Specific activities of enzymes are in μmoles/mg protein/hr.

* Significantly different from control.

† Significantly different at p < 0.05 from KBrO_3_-treated group.

ǂ Significantly different at p < 0.05 from KBrO_3_ and taurine+KBrO_3_ treated group.

LAP: leucineaminopeptidase; AP: alkaline phosphatase; GGT: γ-glutamyltransferase.

The kinetic parameters, K_M_ (Michaelis constant) and V_max_ (maximal velocity), of the enzymes were then determined in isolated BBMV by the double reciprocal Lineweaver-Burk plots. Treatment with KBrO_3_ led to significant lowering of V_max_ values of all BBM enzymes (Table 3). However, prior administration of taurine led to a significant recovery in the V_max_ values. As expected, treatment with taurine alone did not alter V_max_ of any of the four enzymes. The K_M_ values of the enzymes were not altered by any of these treatments and were insignificantly different among the four groups.

**Table 3:** Effect of taurine pre-treatment on KBrO_3_-induced changes in the kinetic parameters of BBM enzymes in purified BBMV.

Groups	K_M_ (mM)	V_max_ (μmoles/mg protein/h)
**LAP**		
Control	0.11±0.002	12.27±1.38
KBrO_3_ alone	0.09±0.001	6.14±0.47*
Taurine alone	0.11±0.002	12.11±1.16ǂ
Taurine+KBrO_3_	0.12±0.001	9.86±0.77* †
**AP**		
Control	32.12±2.21	42.07±3.35
KBrO3 alone	30.98±1.97	30.12±2.79*
Taurine alone	32.48±2.27	41.84±4.31ǂ
Taurine+KBrO_3_	31.79±2.01	39.87±3.86†
**GGT**		
Control	1.88±0.09	11.12±1.18
KBrO_3_ alone	1.76±0.04	6.45±0.17*
Taurine alone	1.66±0.03	11.69±1.11ǂ
Taurine+KBrO_3_	1.71±0.02	9.42±1.04^b^ * †
**Sucrase**		
Control	39.78±2.74	180.68±7.51
KBrO_3_ alone	40.01±3.01	92.33±5.47*
Taurine alone	39.64±2.86	181.13±9.42ǂ
Taurine+KBrO_3_	38.69±2.47	154.27±6.83* †

K_M_ and V_max_were calculated from double reciprocal (1/v vs 1/[S]) Lineweaver-Burk plots. Results are mean ± SEM of four different preparations.

* Significantly different from control.

† Significantly different at p < 0.05 from KBrO_3_-treated group.

ǂ Significantly different at p < 0.05 from KBrO_3_ and taurine+KBrO_3_ treated group.

^a^ Significantly different from control.

^b^ Significantly different from control and KBrO_3_ alone groups.

LAP: leucineaminopeptidase; AP: alkaline phosphatase; GGT: γ-glutamyltransferase.

### ACP and total ATPase

Treatment of rats with KBrO_3_ alone resulted in significant alterations in the activities of ACP and total ATPase when compared to untreated controls. The activity of ACP, a lysosomal marker enzyme, increased while total ATPase decreased significantly. However, pre-treatment with taurine led to significant amelioration in KBrO_3_-induced alterations in the activities of both enzymes (Table 4). Administration of taurine alone had no effect on the activities of these enzymes which were similar to the control group.

**Table 4:** Effect of taurine pre-treatment on KBrO_3_-induced changes in the activities of ACP and total ATPase in intestinal homogenates.

	Control	KBrO_3_ alone	Taurine alone	Taurine+KBrO_3_
ACP	1.79±0.09	4.69±0.16*	1.74±0.05^ǂ^	2.86±0.09* ^†^
Total ATPase	8.14±0.42	4.25±0.11*	8.34±0.41^ǂ^	6.29±0.28* ^†^

Results are mean ± SEM of six different preparations.

Specific activities of enzymes are in μmoles/mg protein/hr.

* Significantly different from control.

† Significantly different at p < 0.05 from KBrO_3_-treated group.

ǂ Significantly different at p < 0.05 from KBrO_3_ and taurine+KBrO_3_ treated group.

ACP: acid phosphatase.

### Parameters of oxidative stress

AO status is a potential biomarker to determine the physiological state of the cell, tissue or organ. The ROS generated under conditions of OS cause increase in LPO, protein oxidation as well as oxidation of protein and non-protein (mainly GSH) sulfhydryl groups. These parameters were determined in intestinal homogenates prepared from animals in the four groups. LPO was determined from levels of malondialdehyde which reacts with thiobarbituric acid to give a pink coloured product. Protein oxidation increases carbonyl groups which were determined after reaction with 2,4-dinitrophenyl hydrazine. Administration of KBrO_3_ alone greatly enhanced both LPO and protein oxidation as reflected by almost four fold higher levels of TBARS and protein carbonyls compared to the control group. It also resulted in significant reduction in total SH and GSH content (Table 5). There was also a marked increase in the level of H_2_O_2_, an ROS, in intestinal homogenates in KBrO_3_ treated animals. However, these KBrO_3_-induced changes were significantly attenuated by administration of taurine prior to treatment with KBrO_3_ while treatment with taurine alone did not significantly alter any of these parameters. These results indicate marked protection by taurine against KBrO_3_ induced OS in the intestinal tissue (Table 5).

**Table 5:** Effect of taurine pre-treatment on KBrO_3_-induced changes in some parameters of oxidative stress in intestinal homogenates.

	Control	KBrO_3_ alone	Taurine alone	Taurine+KBrO_3_
TBARS	41.39±2.41	162.61±7.82*	38.59±2.09^ǂ^	71.67±4.01* ^†^
Carbonyl	60.81±4.17	234.85±10.01*	57.96±3.28^ǂ^	110.64±8.51* ^†^
Total SH	13.25±1.30	5.39±0.24*	13.87±1.73^ǂ^	10.92±1.17* ^†^
GSH	1.98±0.13	0.76±0.07*	2.03±0.12^ǂ^	1.57±0.11* ^†^
H_2_O_2_	129.22±8.14	329.54±14.86*	105.22±8.01* ^ǂ^	182.88±8.64* ^†^

Results are mean±SEM of six different preparations. TBARS, carbonyl content and H_2_O_2_ levels are in nmoles/g tissue while total SH and GSH are in μmoles/g tissue.

* Significantly different from control.

† Significantly different at p < 0.05 from KBrO_3_-treated group.

ǂ Significantly different at p < 0.05 from KBrO_3_ and taurine+KBrO_3_ treated group.

TBARS: thiobarbituric acid reactive substances; SH: sulfhydryl; H_2_O_2_: hydrogen peroxide GSH: glutathione.

### Antioxidant enzymes

The effect of pre-treatment with taurine on KBrO_3_-induced alterations in the activities of some AO enzymes in mucosal homogenates was determined (Table 6). Administration of KBrO_3_ alone to rats caused marked changes in the activities of major detoxifying enzymes like SOD, CAT and GPx. It resulted in a decline in the activities of GR and TR while GST was significantly enhanced. However, these KBrO_3_-induced changes in AO enzyme activities were significantly attenuated by prior administration of taurine. The results show taurine protects the cellular enzymatic AO defence of intestinal mucosal tissue from alterations induced by KBrO_3_ (Table 6).

**Table 6:** Effect of taurine pre-treatment on KBrO_3_-induced changes in the activities of some anti-oxidant enzymes in intestinal homogenates.

	Control	KBrO_3_ alone	Taurine alone	Taurine+KBrO_3_
CAT	12.86±1.21	4.22±0.86*	12.94±1.18^ǂ^	9.54±1.01* ^†^
SOD	78.66±3.47	173.68±7.01*	79.04±3.28^ǂ^	97.84±4.01* ^†^
GPx	4.12±0.54	1.71±0.18*	4.38±0.31^ǂ^	3.21±0.28* ^†^
GST	12.97±1.86	31.68±2.74*	13.01±0.12^ǂ^	16.37±2.11* ^†^
GR	23.47±2.01	8.56±1.01*	23.89±2.19^ǂ^	18.87±1.64* ^†^
TR	7.78±0.89	2.09±0.08*	7.94±0.91^ǂ^	5.88±0.26* ^†^

Results are mean±SEM of six different preparations. Specific activity of SOD is in units/mg protein (One unit is the amount which causes 50% inhibition of pyrogallol auto-oxidation in a reaction volume of 3 ml). Specific activities of CAT, GPx, GST, GR and TR are in nmol/mg protein/min.

* Significantly different from control.

† Significantly different at p < 0.05 from KBrO_3_-treated group.

ǂ Significantly different at p < 0.05 from KBrO_3_ and taurine+KBrO_3_ treated group.

CAT: catalase; SOD: Cu-Zn superoxide dismutase; GPx: glutathione peroxidase; GST: glutathione-S-transferase; GR: glutathione reductase; TR: thioredoxin reductase.

### Carbohydrate metabolism

The activities of enzymes of several metabolic pathways were determined in mucosal homogenates (Table 7). These include LDH (glycolysis), MDH (citric acid cycle), G6P and FBP (gluconeogenesis), G6PD (pentose phosphate pathway) and ME (NADPH generation). KBrO_3_ treatment significantly increased the activity of LDH while MDH was decreased (Table 7). It also decreased the activities of gluconeogenic enzymes, G6P and FBP. The effect of KBrO_3_ was also determined on G6PD and ME that are the major source of NADPH which is needed in various anabolic reactions. Treatment with KBrO_3_ alone significantly decreased G6PD but increased ME activity. However, in the taurine+KBrO_3_ group there was significant attenuation in the KBrO_3_-induced alterations in the activities of all these metabolic enzymes (Table 7). Thus taurine restores the metabolic pathways that were altered by exposure to KBrO_3_. Administration of taurine alone did not significantly alter the activities of any of these enzymes.

**Table 7:** Effect of taurine pre-treatment on KBrO_3_-induced changes in the activities of enzymes of carbohydrate metabolism in intestinal homogenates.

	Control	KBrO_3_ alone	Taurine alone	Taurine+KBrO_3_
LDH	13.81±2.14	47.70±3.42^a^ *	14.55±1.58^ǂ^	17.01±2.04* ^†^
MDH	11.42±1.13	4.09±0.35^a^ *	11.77±1.07^ǂ^	9.48±1.01* ^†^
ME	1.18±0.12	16.08±1.96^a^ *	1.57±0.22^ǂ^	4.15±0.48* ^†^
FBP	1.01±0.09	0.51±0.04^a^ *	1.07±0.06^ǂ^	0.90±0.02* ^†^
G6P	0.31±0.05	0.16±0.01^a^ *	0.35±0.04^ǂ^	0.26±0.02* ^†^
G6PD	6.64±1.19	1.01±0.08^a^ *	6.92±1.13^ǂ^	5.24±1.01* ^†^

Results are mean±SEM of six different preparations. Specific activities of LDH, MDH, ME and G6PD are in nmoles/mg protein/min while FBP and G6P are in μmoles/mg protein/hr.

* Significantly different from control.

† Significantly different at p < 0.05 from KBrO_3_-treated group.

ǂ Significantly different at p < 0.05 from KBrO_3_ and taurine+KBrO_3_ treated group.

LDH: lactate dehydrogenase; MDH: malate dehydrogenase; ME: malic enzyme; FBP: fructose 1,6-bisphosphatase; G6P: glucose 6-phosphatase; G6PD: glucose 6-phosphate dehydrogenase.

### Comet assay

**Fig 1:**
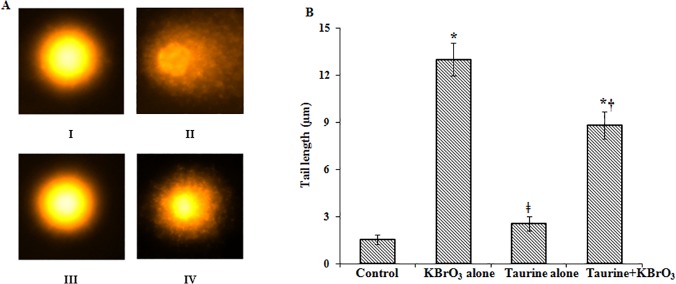
Comet assay of intestinal mucosal cells to assess the degree of DNA breakage. (A) Mucosal cells were subjected to alkaline single cell gel electrophoresis (comet assay) and visualized under a fluorescent microscope after staining the DNA with ethidium bromide. I: Control; II: KBrO_3_ alone; III: taurine alone; IV: taurine+KBrO_3_. (B) Comet tail lengths. The results of comet assay in panel (A) are represented as mean values of tail lengths. *Significantly different at p < 0.05 from control. † Significantly different at p < 0.05 from KBrO_3_-treated group. ǂ Significantly different at p < 0.05 from KBrO_3_ and taurine+KBrO_3_ treated group.

Treatment of rats with KBrO_3_ alone caused significant DNA damage in intestinal mucosa when compared to untreated control. This was evident from the elongated tail length, indicating increased level of single strand breaks and alkali labile sites in mucosal DNA (Fig. 1). Pre-treatment with taurine reduced the extent of KBrO_3_-induced DNA damage resulting in decrease in comet tail length. Taurine by itself did not cause DNA damage in the mucosal tissue and the comet tail length was almost the same as in control cells.

### DNA fragmentation and DPC

**Fig 2:**
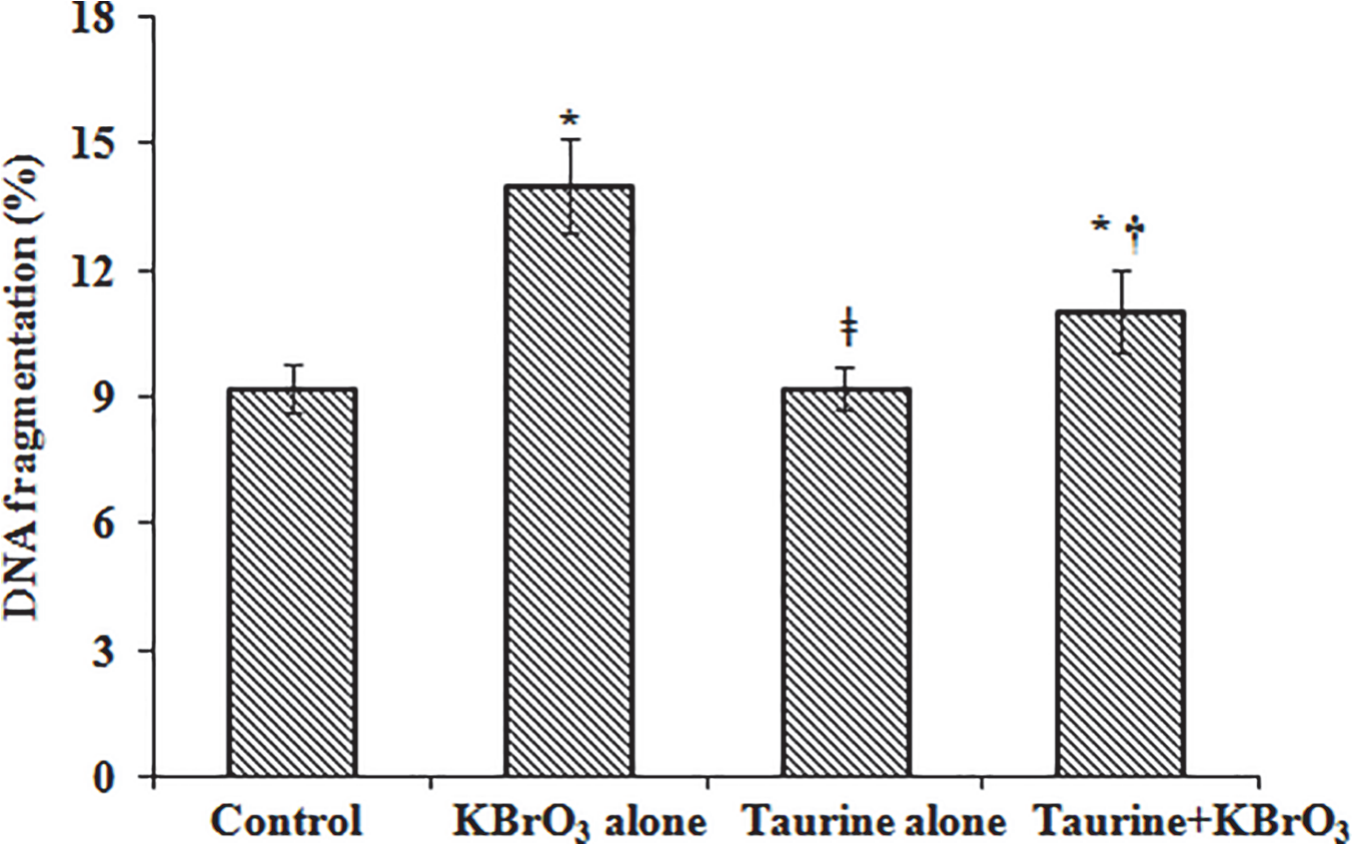
DNA fragmentation in intestinal mucosal homogenates determined by diphenylamine assay. Results are mean ±SEM of 6 different preparations. * Significantly different at *p*< 0.05 from control. † Significantly different at p < 0.05 from KBrO_3_-treated group. ǂ Significantly different at p < 0.05 from KBrO_3_ treated group

Oral administration of KBrO_3,_ led to significant induction of DPC in intestinal mucosal homogenates (∼3-fold) when compared to the untreated control group (Table 8). It also induced DNA fragmentation (∼2-fold) with release of nucleotides in the supernatant as measured by the diphenylamine assay. Pre-treatment with taurine significantly decreased KBrO_3_-induced DNA fragmentation and DPC in intestinal mucosa while taurine alone had no effect (Fig. 2).

**Table 8:** Effect of taurine pre-treatment on KBrO_3_-induced DPC in intestinal mucosal homogenates.

	Protein cross linked DNA %^a^	DPC coefficient^b^
Control	2.65±0.11	1
KBrO_3_ alone	7.88±1.05*	2.97
Taurine alone	2.93±0.19^ǂ^	1.10
Taurine+KBrO_3_	4.67±0.53* ^†^	1.76

Results are mean±SEM of six different preparations.

^a^ Protein cross-linked DNA/ total DNA.

^b^ Ratio of protein cross-linked DNA (%) in treated animals to protein cross-linked DNA (%) in control animals

* Significantly different from control.

† Significantly different at p < 0.05 from KBrO_3_-treated group.

ǂ Significantly different at p < 0.05 from KBrO_3_ and taurine+KBrO_3_ treated group.

DPC: DNA-protein cross-links.

### Histology

**Fig 3:**
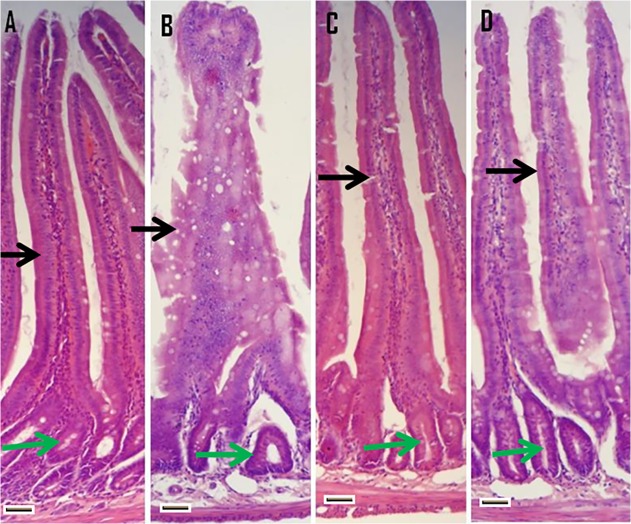
Histology of hematoxylin and eosin stained sections of rat intestine (duodenum section). Duodenum in the untreated control (A) shows normal epithelia of both villi and intestinal glands whereas KBrO_3_ treated group (B) reveals extensive damage of both components with the lumen being filled with debris. The taurine alone group (C) shows reasonably well preserved condition of both components while taurine+KBrO_3_ treated group (D) shows resumption in the morphology of both villi and intestinal glands. Intestinal villi are shown by black arrow and intestinal crypts by green arrow. Magnification is 200 X, scale bar [−] = 50 μm.

Histological examination of the duodenum from control animals revealed normal appearance of villi, brush border bearing enterocytes and intestinal crypts containing different types of cells with clear lumen. Marked histological changes were seen in the duodenum of KBrO_3_-treated rats and extensive intestinal damage was observed. The normal contour of the villi was lost in the form of swelling, oedema and deformation. There was obvious loss and disorganization of surface enterocytes with loss of brush border and infiltration by numerous inflammatory cells. The villus surface is laden with mucus filled goblet cells imparting a vacuolated appearance most obvious in the middle of the villus (Fig. 3). Pre-treatment with taurine greatly attenuated the tissue damage induced by KBrO_3_. As expected, the taurine alone group showed reasonably well preserved condition of both the components and thus this amino acid does not induce any lesions in the intestine of rats (Fig. 3).

## Discussion

The small intestine is exposed on a continuous basis to high levels of ROS and requires a strong AO defence system, which may preserve its endocrine, metabolic, digestive and absorptive functions [42]. This is important since redox disturbances are known to negatively impact body systems through the generation of ROS, which can modify proteins, lipids, and DNA. Research during the past two decades has identified some of the key events that are involved in mucosal damage and AO defence mechanism [42–44]. Thus, it is important to identify nutrients and/or AO capable of counteracting the detrimental effect of various toxicants’ induced OS and associated pathologies. Taurine is one such dietary compound which is found in several foods and has been shown to reduce the risk of various disorders. It exhibits protective effects against several environmental agents due to its well known AO, antimutagenic and anticarcinogenic properties. The aim of this study was to investigate whether taurine could abrogate the KBrO_3_-induced oxidative damage to rat intestine.

The BBM lining the epithelial cells of small intestine is one of the most important cellular membranes, owing to its role in the digestion and absorption of nutrients. This process of digestion and absorption can be altered by drugs, chemicals, nutritional status and toxic pollutants [42]. A decrease in the activities of BBM enzymes was seen upon administration of KBrO_3_. This could be due to the direct modification and consequent inactivation of these enzymes by KBrO_3_ generated free radicals and ROS. There could also have been leakage or loss of these enzymes into the lumen of the intestine following ROS induced damage to the epithelial cells, especially the membrane. Increase in LPO, which affects membrane structure and function, could also have resulted in a decrease in the activities of these enzymes. The administration of taurine prior to treatment with KBrO_3_ greatly reverted the activities of these enzymes. Since KBrO_3_ is well known to induce OS in the cell [5,45], the protective effect of taurine on KBrO_3_-induced enzyme inactivation could have been due to the effectiveness of taurine in inhibiting the chain reactions of KBrO_3_ generated free radicals before they reached their cellular targets. The consequent reduction in LPO and oxidative modification of BBM enzymes might have contributed to the efficacy of taurine in attenuating the effects of KBrO_3_. The administration of taurine alone did not lead to significant alterations in the activities of BBM enzymes and they were insignificantly different from the control values. Kinetic studies, using isolated BBMV, showed that the decrease in activities of these enzymes was due to change in V_max_ values. The K_M_ remained the same showing that the affinity of BBM enzymes for their substrates remains the same upon administration of taurine, KBrO_3_ or taurine plus KBrO_3_. The activity of lysosomal enzyme ACP was significantly increased in the intestinal homogenates by administration of KBrO_3_, which could result in lysosome mediated cell damage.

A major function of small intestine is to absorb important ions and molecules, which in turn depends on the structural integrity and available energy supplied by various metabolic pathways. Thus, it is possible that any alterations in these metabolic pathways caused by toxicants would affect the function of the small intestine. To assess the metabolic aspects, the activities of various enzymes of carbohydrate metabolism were determined. The activities of enzymes of glycolysis, tricarboxylic acid cycle, gluconeogenesis and hexose monophosphate shunt pathway were differentially altered in intestinal tissue by KBrO_3_ treatment. The administration of KBrO_3_ alone caused significant increase in LDH and decrease in MDH in the intestinal mucosal cells. The administration of KBrO_3_ alone caused differential effect on the enzymes of gluconeogenesis and hexose monophosphate shunt pathway. The activity of G6P and FBP significantly decreased whereas the activity of G6PD and ME increased profoundly upon KBrO_3_ treatment. Administration of taurine prior to KBrO_3_ treatment resulted in an overall improvement of carbohydrate metabolism as evident by increased activities of LDH, MDH and gluconeogenic enzymes. Taurine might have lowered the number of damaged mitochondria or affected macromolecules or may have increased number of normally active organelles or macromolecules. Taurine due to its anti-inflammatory property might have reduced organ damage and subsequent cell mortality.

The imbalance between free radical production and scavenging ability is thought to lead to ROS generation and cellular injury. One of the deleterious effects of ROS is the oxidation of lipids and proteins, which are important components of biomembranes. In agreement with previous results [5], KBrO_3_ significantly enhanced LPO and protein carbonyls in intestinal mucosal tissue suggesting the induction of OS. The level of H_2_O_2_, an ROS, was significantly increased. H_2_O_2_ is a strong oxidant which can also generate the damaging hydroxyl radical upon reaction with transition metal ions in the cell. The level of GSH, the most abundant low molecular weight thiol which acts as a physiological AO in the cell, and total thiols were decreased. However, prior administration of taurine greatly lowered the KBrO_3_-induced OS as evident by significantly recovered values of LPO and carbonyls along with total thiol levels. H_2_O_2_ and GSH levels were also greatly restored to normal values compared to those in the KBrO_3_ alone group. Taurine either stabilises the lipid membranes or protects them from the damaging peroxides. Besides taurine is also known to stimulate the synthesis of GSH and G6PD, and hence can effectively reduce the KBrO_3_-induced OS in intestinal tissue. Similar protective effects of taurine on intestinal damage have been reported earlier by other workers with other agents [46,47].

Small intestine is one of the major targets of ROS that are generated by orally ingested xenobiotics like KBrO_3_ and responds to such toxic insult by altering the activities of various AO defence enzymes [2,5,45]. KBrO_3_ treatment resulted in significant alterations in the activities of AO enzymes like CAT, SOD, GPx, GR, GST and TR. However pre-treatment with taurine produced a significant protection against KBrO_3_-induced changes in AO enzymes by restoring them to control values. Taurine might have either stimulated the activity of these AO enzymes or itself neutralised the excess ROS generated by KBrO_3_. These observations support the hypothesis that the mechanism of toxicity is related to free radical generation and the chemoprevention offered by taurine is due to free radical scavenging property as reported previously by [18].

Oral administration of KBrO_3_ induced DNA degradation in intestinal tissue of treated animals. This was seen in the comet assay which showed increased level of DNA single strand breaks and alkali labile sites in the mucosal cells of the intestine. DNA-protein cross-links which impede the activities of proteins involved in DNA replication, transcription and repair, were also significantly enhanced in KBrO_3_ treated rats [6]. The pre-treatment of animals with taurine reduced the level of KBrO_3_-induced DNA damage and DPC formation probably by its anti-mutagenic action as has been reported earlier in other systems [48–50].

Histological observations of the duodenum strongly support the biochemical results. The intestine from KBrO_3_ treated animals showed extensive intestinal damage. The lumen was full of debris, inflammatory cells and intestinal villi had lost their contour and prominence of mucus secreting cells was observed, both in the remnant of villi as well as in the intestinal crypts. These changes were greatly reduced by prior administration of taurine, probably by neutralising the excessive formation of ROS and thereby reducing the morphological and cellular damage.

The protection offered by taurine against KBrO_3_-induced gastrointestinal toxicity as seen here is probably due to the diminution in KBrO_3_-induced OS due to its AO property. Unlike polyphenolic antioxidants, taurine itself does not directly quench classical ROS and free radicals like H_2_O_2_, superoxide anion and hydroxyl radical but its metabolic precursor, hypotaurine, has been shown as an efficient radical scavenger. Taurine is widely believed to act as an AO for which several reasons have been proposed [51–56]. First, taurine regulates the levels of various endogenous AO. It enhances the synthesis of GSH and also stimulates the activity of G6PD, an enzyme that generates NADPH which is required by glutathione reductase to convert oxidised glutathione into GSH. Second, taurine protects cells from injury and subsequent necrosis by preventing Ca^2+^ overload via Na^2+^/Ca^2+^ exchanger. Third, taurine conjugates with MDA, the end product of LPO, thereby stabilising the lipid bilayer and making the membranes lipids less vulnerable to ROS induced toxic insult. Fourth, due to its strongly negatively charged sulfonic group, taurine can conjugate redox active metals like Fe^2+^ and Cu^2+^, reducing their reaction with H_2_O_2_, a process which generates the damaging hydroxyl radical. Fifth, taurine has been shown to prevent the diversion of electrons from respiratory chain to other acceptors such as oxygen, thereby inhibiting the accumulation of ROS like superoxide anion. Finally, taurine can act as an organic osmolite by increasing tissue osmolarity and conferring more flexibility to the cell membrane. Either one or more of these mechanisms may be involved in protecting the intestine from toxicity induced by KBrO_3_.

**Fig 4:**
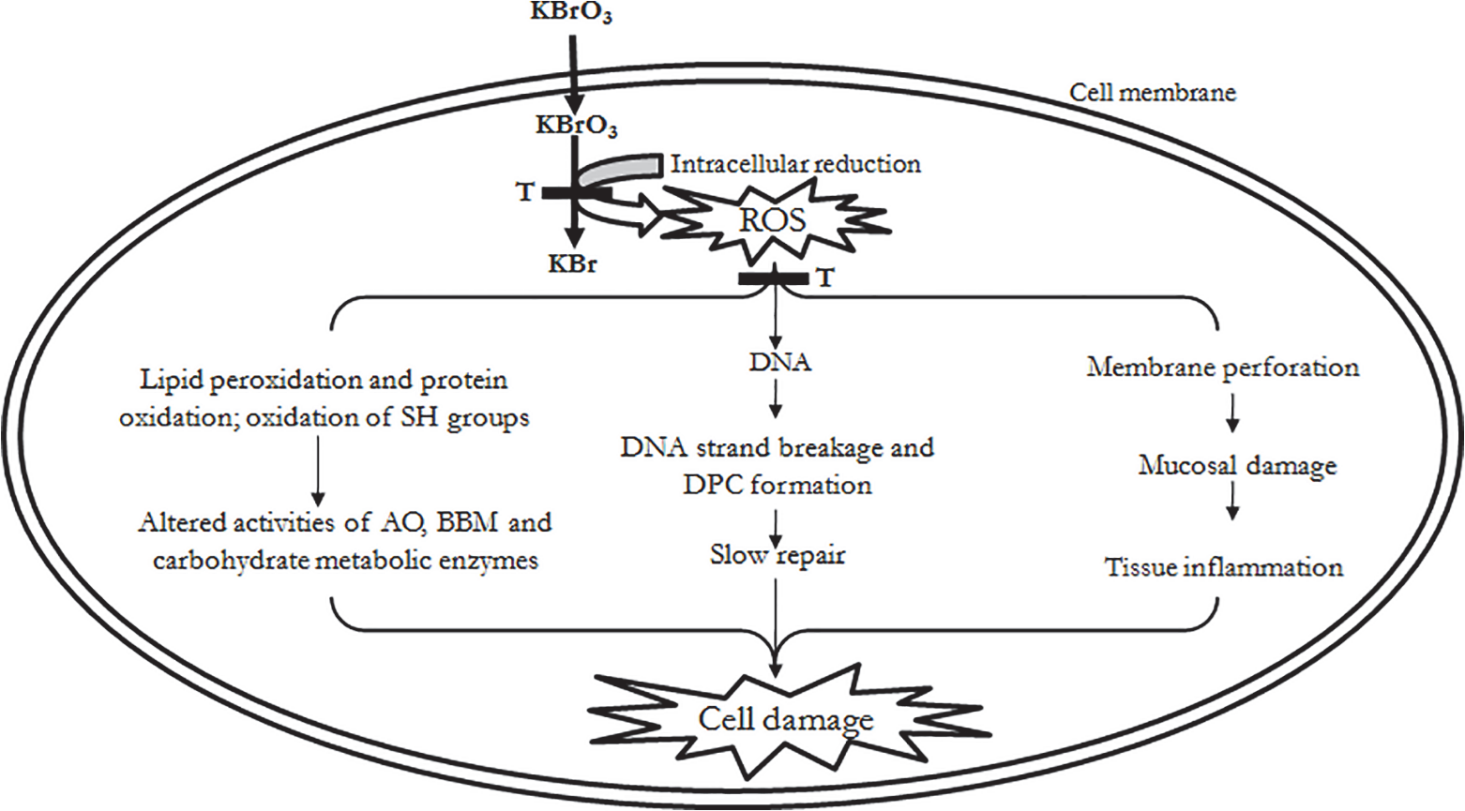
Chemoprotective effects of taurine on KBrO_3_ induced alterations in intestinal cell metabolism, membrane integrity and oxidative stress: a summary. AO: antioxidant; BBM: brush border membrane; DPC: DNA-protein cross-links; KBr: potassium bromide; KBrO_3_: potassium bromate; ROS: reactive oxygen species; SH: sulfhydryl; T: taurine.

In summary, our results show that OS induced by KBrO_3_ contributes to the development of intestinal toxicity. The consequences are dramatic alterations in various cellular/physiological processes. Pre-treatment with taurine greatly reverses KBrO_3_-induced tissue damage suggesting that it may be beneficial to intestinal function during free radical attack. A general scheme, based on our experimental data, summarizes the KBrO_3_-induced changes in the small intestine and their attenuation by taurine (Fig. 4). However, further studies are warranted to elucidate the exact mechanism of protective effect of taurine on KBrO_3_-induced gastrointestinal tract dysfunction.
